# Distinct Chemokine Dynamics in Early Postoperative Period after Open and Robotic Colorectal Surgery

**DOI:** 10.3390/jcm8060879

**Published:** 2019-06-19

**Authors:** Malgorzata Krzystek-Korpacka, Marek Zawadzki, Paulina Lewandowska, Krzysztof Szufnarowski, Iwona Bednarz-Misa, Krzysztof Jacyna, Wojciech Witkiewicz, Andrzej Gamian

**Affiliations:** 1 Department of Medical Biochemistry, Wroclaw Medical University, 50-368 Wroclaw, Poland; p.lewandowska2803@gmail.com (P.L.); iwona.bednarz-misa@umed.wroc.pl (I.B.-M.); gamian@iitd.pan.wroc.pl (A.G.); 2 Department of Oncological Surgery, Regional Specialist Hospital, 51-124 Wroclaw, Poland; zawadzki@wssk.wroc.pl (M.Z.); jacyna@wssk.wroc.pl (K.J.); witkiewicz@wssk.wroc.pl (W.W.); 3 Infection Control Unit, Regional Specialist Hospital, 51-124 Wroclaw, Poland; szufnarowski@wssk.wroc.pl; 4 Research and Development Centre at Regional Specialist Hospital, 51-124 Wroclaw, Poland; 5 Laboratory of Medical Microbiology, Ludwik Hirszfeld Institute of Immunology and Experimental Therapy, Polish Academy of Sciences, 53-114 Wroclaw, Poland

**Keywords:** surgical stress response, surgical site infection, Th1/Th2 balance, interleukin-8, monocyte chemoattractant protein-1 (MCP1), postoperative ileus, anastomotic leak, robotic surgery, minimally invasive surgery

## Abstract

Stress response to robot-assisted colorectal surgery is largely unknown. Therefore, we conducted a prospective comparative nonrandomized study evaluating the perioperative dynamics of chemokines: IL-8/CXCL8, MCP-1/CCL2, MIP-1α/CCL3, MIP-1β/CCL4, RANTES/CCL5, and eotaxin-1/CCL11 in 61 colorectal cancer patients following open colorectal surgery (OCS) or robot-assisted surgery (RACS) in reference to clinical data. Postoperative IL-8 and MCP-1 increase was reduced in RACS with a magnitude of blood loss, length of surgery, and concomitant up-regulation of IL-6 and TNFα as its independent predictors. RANTES at 8 h dropped in RACS and RANTES, and MIP1α/β at 24 h were more elevated in RACS than OCS. IL-8 and MCP-1 at 72 h remained higher in patients subsequently developing surgical site infections, in whom a 2.6- and 2.5-fold increase was observed. IL-8 up-regulation at 24 h in patients undergoing open procedure was predictive of anastomotic leak (AL; 94% accuracy). Changes in MCP-1 and RANTES were predictive of delayed restoration of bowel function. Chemokines behave differently depending on procedure. A robot-assisted approach may be beneficial in terms of chemokine dynamics by favoring Th1 immunity and attenuated angiogenic potential and postoperative ileus. Monitoring chemokine dynamics may prove useful for predicting adverse clinical events. Attenuated chemokine up-regulation results from less severe blood loss and diminished inflammatory response.

## 1. Introduction

Surgical intervention evokes a stress response in immunological systems. If deficient, it may contribute to immunosuppression and increased susceptibility to infections. If excessive, it may lead to systemic inflammatory response syndrome with subsequent organ failure [1]. In cancer, inflammatory response and immunosuppression following surgery additionally create an environment that potentially facilitates the growth and spread of dormant micrometastases or circulating tumor cells [2]. As the magnitude of stress response is directly proportional to the extent of trauma, a minimal invasive surgery (MIS) is increasingly employed [1]. Robotic surgery is a step beyond traditional laparoscopy. However, while advantageous in many aspects, it is associated with unfavorably longer operating times, prolonged anesthesia, and suboptimal position of the body. An attenuated surgical stress following laparoscopic colorectal surgery has been shown, but the effects of the robot-assisted approach remain poorly explored.

Chemokines are secreted by endothelial, stromal and immune cells in response to inflammatory and stress signals, and play critical role in the trafficking and positioning of all leukocytes, facilitating their rapid movement to the site of injury [3]. Yet, the impact of a surgical approach on their perioperative dynamics in colorectal surgery has not been addressed. Here, we characterized the temporal changes in chemokines to shed some light on the body response to robot-assisted colorectal surgery as compared with the classic open procedure. We also aimed to identify predictors of initial chemokine rise/drop from among various patient- and surgery-related parameters. Additionally, to evaluate clinical relevance of differences in chemokine dynamics, we assessed their association with adverse outcomes, such as surgical site infections, anastomotic leak (AL), and prolonged hospital stay and restoration of bowel function, which are known to be improved through a robotic approach.

## 2. Experimental Section

### 2.1. Patients

This prospective, comparative, nonrandomized study was conducted within the WROVASC Integrated Cardiovascular Centre project. The study enrolled 61 patients with colorectal cancer undergoing curative resection in the Department of Surgical Oncology, Regional Hospital in Wroclaw, during the years 2013–2015. Routine preoperative workup included colonoscopy, abdominal and pelvic computed tomography, and pelvic magnetic resonance imaging for rectal cancer. Patients’ physical status was expressed in accordance with the American Society of Anesthesiologists (ASA) classification. Exclusion criteria were: age < 18 years, ASA > 3, emergency surgery, gross metastatic disease, locally advanced cancers not amenable to curative resection, tumors requiring en bloc multi-visceral resection, coexisting malignancies, severe cardiovascular or respiratory disease, diabetes mellitus, severe mental disorders, and immunological diseases requiring systemic administration of corticosteroids. The tumor-node-metastasis (TNM) Staging System devised by the Union for International Cancer Control (UICC) was used to determine the stage of neoplastic disease and the Clavien-Dindo Classification was used to assess complications [4].

Decision on open colorectal surgery (OCS) or robot-assisted colorectal surgery (RACS) using the Da Vinci Si console (Intuitive Surgical Sunnyvale, CA, USA) was undertaken by the patient after discussion with a surgeon. All the robotic procedures in this study were performed by two colorectal surgeons with credentials in robotic surgery who performed at least 30 robotic operations prior to this study initiation. 

All patients received mechanical bowel preparation, perioperative antibiotic and low molecular weight-heparin prophylaxis. Parenteral opioids were used to control postoperative pain. Surgical drains were routinely used and removed on the 1st/2nd postoperative day. Restoration of bowel function (RoBF) was defined as tolerance of solid diet and passage of first stool.

Documentation on surgical site infection (SSI, defined using Centers for Disease Control and Prevention criteria [5]) was collected prospectively by surgical nurse (and surgeon whenever in hospital) and by trained infection control personnel via telephone survey within 30 days after the surgery. Data on white blood cells (WBC), neutrophils (NEU), and lymphocytes (LYM) were available preoperatively for all patients and at 24 h after surgery for 54 patients. Anemia was defined as hemoglobin < 12 g/dL in women and <13.5 g/dL in men. Overweight/obesity was defined as having body mass index (BMI) ≥ 25. Data on BMI was available for 59 patients. Characteristics of the study population, in part described previously in [6,7], are given in Table 1.

Blood for chemokine analysis was collected prior to surgery and at 8, 24, and 72 h after the incision.

### 2.2. Ethical Considerations

The study protocol was approved by the Medical Ethics Committees of Regional Specialist Hospital (#KB/nr 1/rok 2012 from 26 June 2012) and the study was conducted in accordance with the Helsinki Declaration of 1975, as revised in 1983. Informed consent was obtained from all patients. 

### 2.3. Analytical Methods

Blood was drawn by venipuncture, clotted (30 min) and centrifuged (15 min, 720 × g). Sera were stored at −80 °C. Interleukin-8 (CXCL8), macrophage inflammatory proteins (MIP)-1α (CCL3) and -1β (CCL4), monocyte chemoattractant protein (MCP)-1 (CCL2), eotaxin 1 (EOX1/CCL11), were regulated on activation. Normal T cells that were expressed and secreted (RANTES/CCL5) were selected from the group I cytokine panel (#m500kcaf0y) of Bio-Plex Pro Human Cytokine/Chemokine/Growth Factor Magnetic Bead–Based Assays and measured in duplicates/triplicates using the BioPlex 200 platform (Bio-Rad, Hercules, CA, USA). Assay sensitivities and intra- and inter-assay coefficients of variation were as follows: 1 pg/mL, 9% and 4% (IL-8); 1.1 pg/mL, 9% and 7% (MCP-1); 1.6 pg/mL, 7% and 8% (MIP-1α); 2.4 pg/mL, 8% and 8% (MIP-1β); 2.5 pg/mL, 8% and 11%; (EOX1), and 1.8 pg/mL, 9% and 6% (RANTES). Data were analyzed using 5-PL logistic regression and BioPlex Manager 6.0 software. 

For the purpose of correlation analysis, IL-1β, TNF-α, and IL-6 were retrieved from our database [6].

### 2.4. Statistical Analysis

Data distribution was tested using a Chi-squared (χ^2^) test, and homogeneity of variation was investigated using Levene tests. Chemokine data required log-transformation, and are presented as geometric means with a 95% confidence interval (CI). Depending on data character, they were analyzed using as *t*-test for independent samples, with Welch correction in cases of unequal variances, or a one-way ANOVA with Tukey-Kramer post hoc test. A two-way ANOVA with repeated measures on one factor (F; time of measurement) was used to evaluate the effect of surgical approach and of other clinical parameters on chemokine dynamics. Patients were stratified into groups (G) based on patient- or surgery-related clinical parameters. Results of those analyses are reported as significance (Bonferroni corrected *p* values) for the effect of factor (F), group (G), and their interaction (F × G). Correlation analysis was conducted using a Pearson test. Two-way ANOVA was used to adjust for the effect of surgery. Logistic and multiple regression (stepwise methods with *p* < 0.05 as an entrance criterion and *p* > 0.1 as a removal criterion) were used to determine independent predictors of initial chemokine elevation (multiple linear regression) or adverse clinical outcomes (logistic regression). Frequency analysis was conducted using a Fisher exact test (2 × 2 tables) or Chi-squared test (2 × 3 and larger). Receiver operating characteristics (ROC) curve analysis was used to determine the accuracy of the devised model/chemokines (expressed in terms of area under curve (AUC) with a 95% CI). The power of the applied tests ranged between 0.7–0.9 for mean comparisons and 0.8–1 for correlation analyses. All calculated probabilities were two-tailed and *p*-values ≤ 0.05 were considered significant. MedCalc Statistical Software version 19.0.3 (MedCalc Software bvba, Ostend, Belgium; https://www.medcalc.org; 2019) was used.

Data were analyzed either as absolute values at a given time point (denoted in a subscript; e.g., MCP-1_24h_) or as fold or percentage change, allowing for quantification of a change in a variable over time. Fold change, denoted as Δ, was calculated as an absolute difference between measurements obtained at two time points (t_1_ and t_2_) and divided by t_1_ (fold change). Fold change multiplied by 100% yielded percentage change. As an example, ΔMCP-1_24h/0_ = 150% expression states that MCP-1 concentration at 24 h post-incision increased by 1.5-fold or 150% as compared with its preoperative level. For each analyzed chemokine, the following fold/percentage changes were calculated: Δ_8h/0_, Δ_24h/0_, Δ_72h/0_, Δ_24h/8h_, Δ_72h/8h_, and Δ_72h/24h_; however, for clarity purposes, only those found significantly different between analyzed groups (OCS vs. RACS, etc.) were reported. 

## 3. Results

Both examined groups were well-matched with respect to age, sex, comorbidities, disease advancement, extent of surgery (expressed in terms of harvested lymph nodes), post-surgical complications and frequency of stomas, but differed in: length of surgery (LoS), which was greater in RACS; estimated blood loss (EBL), which was higher in OCS; SSI, which was more frequent in OCS; and length of hospital stay (LoHS), which was longer in OCS (Table 1).

### 3.1. Chemokine Dynamics and Type of Surgery (Open vs. Robotic)

#### 3.1.1. Interleukin-8 (IL-8/CXCL8)

**Figure 1:**
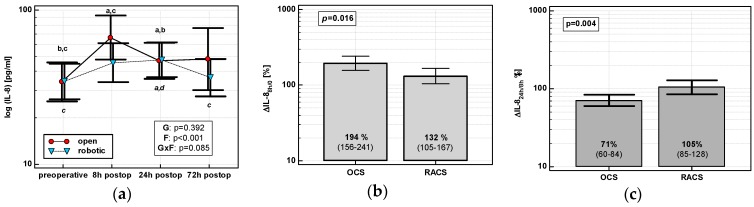
Effect of surgical approach on IL-8. (**a**) Perioperative IL-8 dynamics. Data are presented as geometric means (markers) with a 95% CI (whiskers) and analyzed using two-way repeated measures ANOVA with the statistical significance of group (G), factor (F; time), and their interaction (G × F) effects given in the figure insert. Statistically significant differences between particular time points within open colorectal surgery group (OCS; above marker, straight script) and within robot-assisted surgery group (RACS; below marker, italics) are marked with lower script letters, with “a” denoting preoperative measurement, “b” denoting 8 h, “c” denoting 24 h, and “d” denoting to 72 h. (**b**) Percentage changes in IL-8 between 8 h post-incision and chemokine preoperative concentration (ΔIL-8_8h/0_). (**c**) Percentage changes in IL-8 between 24 and 8 h post-incision (ΔIL-8_24h/8h_). Data are presented as geometric means with a 95% CI and analyzed using a *t*-test for independent samples.

The IL-8 time course displayed a cubic trend in OCS (*p* < 0.0001), but a quadratic one in RACS (*p* = 0.0003). IL-8 in OCS peaked at 8 h and stayed elevated. There was no clear IL-8 peak in RACS, and IL-8 levels returned to baseline at 72 h (Figure 1a). IL-8 up-regulation at 8 h (ΔIL-8_8h/0_) was higher in OCS (by 1.5-fold), followed by a drop at 24 h (ΔIL-8_24h/8h_) (Figure 1b,c).

#### 3.1.2. Monocyte Chemoattractant Protein 1 (MCP-1/CCL2)

**Figure 2:**
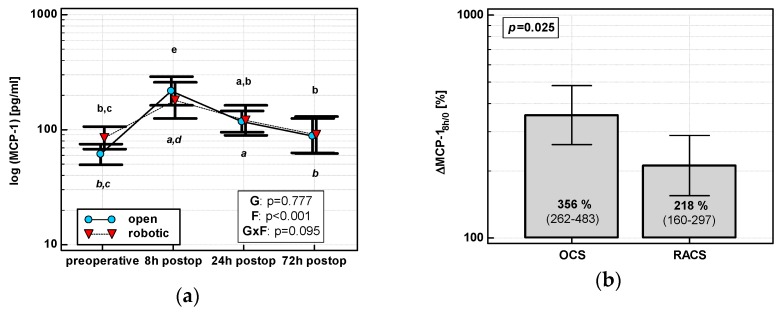
Effect of surgical approach on MCP-1. (**a**) Perioperative MCP-1 dynamics. Data are presented as geometric means (markers) with a 95% CI (whiskers) and analyzed using two-way repeated measure ANOVA with the statistical significance of group (G), factor (F; time), and their interaction (G × F) effects given in the figure insert. Statistically significant differences between particular time points within OCS (above marker, straight script) and RACS (below marker, italics) are marked by lower script letters, with “a” denoting preoperative measurement, “b” denoting 8 h, “c” denoting 24 h, “d” denoting 72 h, and “e” representing all other measurements. (**b**) Relative changes in MCP-1 (ΔMCP-1_8h/0_). Data are presented as geometric means with a 95% CI and analyzed using a *t*-test.

The MCP-1 time course displayed a quadratic trend (*p* < 0.0001) with a peak at 8 h post-incision regardless of surgery type (Figure 2a). As compared with its preoperative level, MCP-1 at 8 h increased by 3.6-fold in OCS and 2.2-fold in RACS, yielding the initial chemokine up-regulation (ΔMCP-1_8h/0_) in OCS to be 1.6 times higher than in RACS (Figure 2b).

#### 3.1.3. Macrophage Inflammatory Protein 1α (MIP-1α/CCL3)

**Figure 3:**
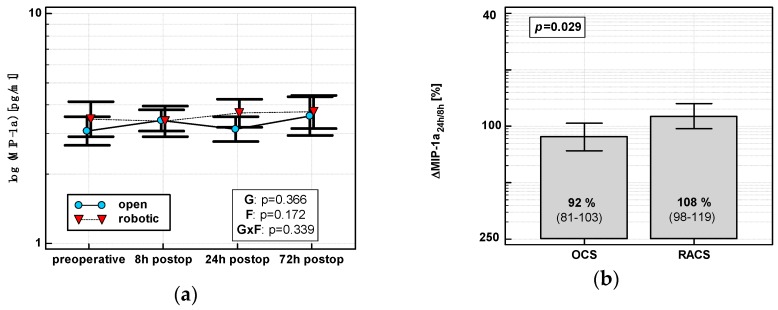
Effect of surgical approach on MIP-1α. (**a**) Perioperative MIP-1α dynamics. Data are presented as geometric means (markers) with a 95% CI (whiskers) and analyzed using two-way repeated measure ANOVA with the statistical significance of group (G), factor (F; time), and their interaction (G × F) effects given in the figure insert. (**b**) Relative changes in MIP-1α (Δ MIP-1α_24h_/_8h_). Data are presented as geometric means with a 95% CI and analyzed using a *t*-test.

In OCS, MIP-1α peaked at 8 h and then dropped. In RACS, MIP-1α peaked at 24 h (Figure 3a). Consequently, ΔMIP-1α24 h/8 h was lower in OCS (Figure 3b).

#### 3.1.4. Macrophage Inflammatory Protein 1β (MIP-1β/CCL4)

**Figure 4:**
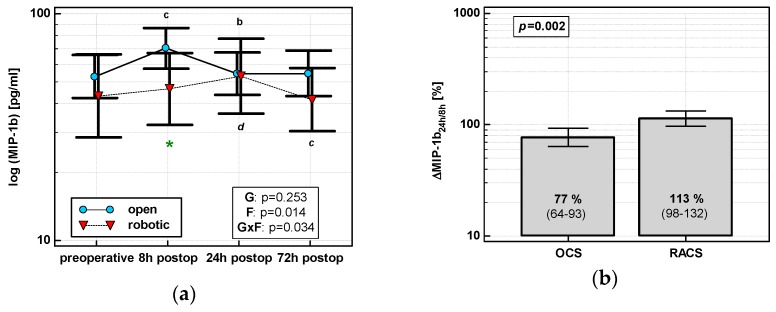
Effect of surgical approach on MIP-1β. (**a**) Perioperative MIP-1β dynamics. Data are presented as geometric means (markers) with a 95% CI (whiskers) and analyzed using two-way repeated measure ANOVA with the statistical significance of group (G), factor (F; time), and their interaction (G × F) effects given in the figure insert. Statistically significant differences between particular time points within OCS (above marker, straight script) and RACS (below marker, italics) are marked by lower script letters with “b” denoting 8 h, “c” denoting 24 h, and “d” denoting 72 h. Significant difference between surgical approaches at a given time point is denoted by a green asterisk. (**b**) Relative changes in MIP-1β (Δ MIP-1β_24h/8h_). Data are presented as geometric means with a 95% CI and analyzed using a *t*-test.

MIP1β levels changed significantly over time, and its time course depended on surgery (Figure 4a). MIP1β displayed a cubic trend (*p* = 0.011) with a maximum at 8 h in OCS and a quadratic trend (*p* = 0.009) with a maximum at 24 h in RACS. Of the evaluated percentage changes, ΔMIP-1β_24h/8h_ was significantly lower in OCS (Figure 4b).

#### 3.1.5. Eotaxin 1 (EOX1/CCL11)

**Figure 5:**
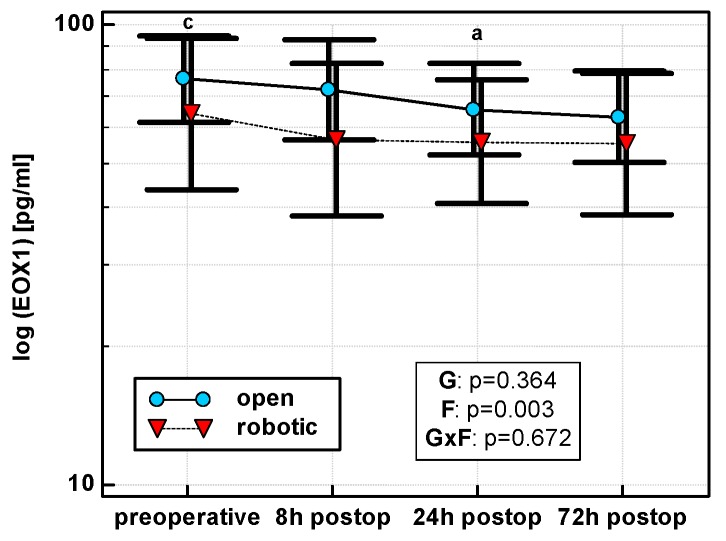
Perioperative eotaxin (EOX1) dynamics. Data are presented as geometric means (markers) with a 95% CI (whiskers) and analyzed using two-way repeated measure ANOVA with the statistical significance of group (G), factor (F; time), and their interaction (G × F) effects given in the figure insert. Statistically significant differences between particular time points within OCS (above marker, straight script) are marked by lower script letters with “a” denoting preoperative measurement, and “c” denoting 24 h.

Eotaxin levels differed with time, displaying a linear decreasing trend in both OCS (*p* = 0.011) and RACS (*p* = 0.049), but surgical approach had no significant effect on mean cytokine levels (Figure 5). Similarly, there were no significant differences between groups in the dynamics of eotaxin when percentage changes were analyzed.

#### 3.1.6. Regulation on Activation, Normal T Cell Expression and Secretion (RANTES/CCL5)

**Figure 6:**
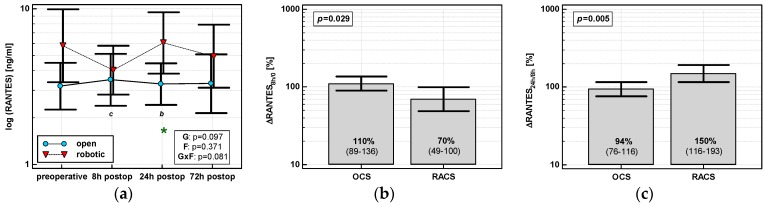
Effect of surgical approach on RANTES. (**a**) Perioperative RANTES dynamics. Data are presented as geometric means (markers) with a 95% CI (whiskers) and analyzed using two-way repeated measure ANOVA with the statistical significance of group (G), factor (F; time), and their interaction (G × F) effects given in the figure insert. Statistically significant differences between particular time points within RACS (below marker, italics) are marked by lower script letters with “b” denoting 8 h, and “c” denoting 24 h. Significant difference between surgical approaches at a given time point is denoted by a green asterisk. (**b**) Relative changes in RANTES (ΔRANTES _8h/0_). Data are presented as geometric means with a 95% CI and analyzed using a *t*-test. (**c**) Relative changes in RANTES (ΔRANTES _24h/8h_). Data are presented as geometric means with a 95% CI and analyzed using a *t*-test.

RANTES concentrations were stable following surgery in OCS, while those in RACS differed and their dynamics displayed a cubic trend (*p* = 0.002) with a drop at 8 h post-incision. A significant between-group difference in RANTES concentrations related to type of surgery was observed at 24 h post-incision (Figure 6a). Percentage changes in chemokine concentrations associated with type of surgery were significant between 8 h post-incision and preoperative level (ΔRANTES _8h/0_), indicative of a drop in RACS and no change in OCS, and between 24 and 8 h post-incision (ΔRANTES _24h/8h_), indicative of a rise in RACS and no change in OCS (Figure 6b,c).

### 3.2. Impact of Other Clinical Parameters on Chemokine Dynamics

The potential effect of clinical parameters other than surgery type on chemokine dynamics was assessed using two-way ANOVA with repeated measures on one factor (time of measurement). Patients were stratified into groups based on patient- or surgery-related clinical parameters. Of the patient-related parameters, the impact of sex, age (dichotomized using 75 years as a cut-off), presence of overweight/obesity or anemia, and general health status expressed in terms of ASA score were examined. Of surgery-related parameters, the effect of surgical procedure (abdominoperineal resection (APR), low anterior resection, right hemicolectomy, left hemicolectomy, and sigmoid resection), estimated blood loss (EBL; dichotomized using a median of 100 mL) and necessity of transfusions, total number of harvested lymph nodes representing extent of the surgery (TNR; dichotomized using a median of 14 nodes), and length of surgery (LoS; dichotomized using a median of 165 min) were evaluated.

#### 3.2.1. Interleukin-8 (IL-8/CXCL8)

None of the evaluated patient-related parameters significantly affected the chemokine time course (*p* values for interaction for all parameters were >0.05). In turn, absolute IL-8 concentration at 8 h was significantly higher in older patients (Supplementary Figure S1).

Of the evaluated surgery-related parameters, EBL significantly affected the chemokine time course (Supplementary Figure S1). An initial elevation in IL-8 was significantly higher (by 1.5-fold) and the drop between 24 and 8 h was more pronounced (by 1.5-fold) in patients with larger EBL (Supplementary Figure S2). Furthermore, surgical procedure significantly affected the chemokine time course. Patients undergoing abdominoperineal resection had steadily increasing IL-8 concentrations while in others surgical procedures the trend was decreasing. However, being based only on two observations, this result must be interpreted with caution (Supplementary Figure S1). 

#### 3.2.2. Monocyte Chemoattractant Protein 1 (MCP-1/CCL2)

Of patient-related parameters, age and BMI significantly affected the MCP-1 time course (interaction *p* < 0.05) (Supplementary Figure S3). Initial chemokine elevation was higher by 2.3-fold in older patients, similar to a higher drop between 24 and 8 h (by 1.9-fold). Overweight/obese individuals had a smaller chemokine drop between 72 and 8 h by 1.9-fold (Supplementary Figure S4).

Of surgery-related parameters, transfusions and LoS significantly affected the chemokine time course and EBL displayed a similar tendency (Supplementary Figure S3). Patients with higher EBL had also higher initial chemokine elevation (by 1.6-fold) and a more pronounced drop between 24 and 8 h (by 1.7-fold). Patients requiring transfusion had increased (and not decreased) chemokine concentrations at 72 h as compared with 24 h, and those with higher LoS had a lower initial chemokine elevation (by 1.7-fold) and less pronounced drop between 24 and 8 h (by 1.8-fold) (Supplementary Figure S4).

#### 3.2.3. Macrophage Inflammatory Protein 1α (MIP-1α/CCL3)

No patient-related parameter significantly affected the chemokine time course, although it tended to differ with respect to patients’ health statuses expressed in terms of ASA (Supplementary Figure S5). Analysis of dynamics-derived measures showed that percentage change in MIP-1α between 8 h and baseline were significantly higher in patients with ASA = 3 as compared to those with ASA = 2. The following drop at 24 h as compared with the baseline could be observed in patients with ASA = 2, while in those with ASA = 1, MIP-1α remained elevated (Supplementary Figure S6).

Of surgery-related parameters, EBL tended to affect the chemokine time course (Supplementary Figure S5) with significant percentage changes between 24 and 8 h (a decrease in higher EBL) and between 72 and 24 h (an increase in higher EBL). LoS was also significantly associated with chemokine percentage change between 72 and 24 h (an increase in lower LoS). Similarly to IL-8, the MIP-1α time course differed in two patients undergoing abdominoperineal resection (Supplementary Figure S6).

#### 3.2.4. Macrophage Inflammatory Protein 1β (MIP-1β/CCL4)

Of patient-related parameters, BMI significantly affected the MIP-1β time course (interaction *p* < 0.05) (Supplementary Figure S7). Analysis of dynamics-derived measures showed that normal-weight patients experienced a drop in chemokine concentration between 72 and 8 h post-incision, which remained stable in overweigh/obese patients. Additionally, a similar observation was made for patients without anemia prior to surgery. In turn, older patients experienced a drop in chemokine concentration at 24 h post-incision as compared to chemokine concentrations at 8 h (Supplementary Figure S8).

Of surgery-related parameters, the time course of MIP-1β concentrations was significantly affected by EBL and tended to be by transfusions and LoS as well (Supplementary Figure S7). Chemokine between 24 and 8 h dropped more markedly in patients with larger EBL and subsequently remained stable, while in patients with smaller EBL it dropped at 72 h as compared with 24 h and not earlier. In patients requiring a transfusion, chemokine was elevated at 72 h as compared with 8 h. Similarly, it was elevated at 24 h as compared with 8 h in patients who were operated on longer (Supplementary Figure S8).

#### 3.2.5. Eotaxin 1 (EOX1/CCL11)

Of patient-related parameters, ASA tended to have an effect (Supplementary Figure S9). As compared with 8 h, chemokine increased at 24 and 72 h in patients with ASA = 1, but decreased in patients with ASA = 2 and 3 (Supplementary Figure S10).

None of the surgery-related parameters significantly affected a time course of eotaxin 1 (Supplementary Figure S9). 

#### 3.2.6. Regulation on Activation, Normal T Cell Expression and Secretion (RANTES/CCL5)

Of patient-related parameters, BMI and anemia prior to surgery tended to have an impact on the chemokine time course (Supplementary Figure S11). RANTES dropped and did not increase at 8 h post-incision in anemic patients, but remained elevated at 72 h. In turn, RANTES concentrations decreased at 72 h in normal-weight patients, but not in overweight/obese patients (Supplementary Figure S12).

Of surgery-related parameters, the RANTES time course was significantly affected by transfusions and tended to be by LoS as well (Supplementary Figure S11). Patients with transfusions had two times higher RANTES at 24 h, and 4.4 times higher at 72 h, as compared with chemokine baseline levels in patients without transfusions. Patients operated on longer had elevated RANTES at 24 and 72 h as compared with 8 h, while in those with lower LoS chemokine concentration decreased (Supplementary Figure S12). 

### 3.3. Predictors of Initial Chemokine Up- or Down-Regulation

The correlation between percentage change in chemokine between 8 h post-incision and preoperative level (Δ_8h/0_) and patients’ age, BMI, Charlson Comorbidity Score (CCS), TNR, LoS, and EBL as continuous variables and relative changes in classic proinflammatory cytokines ΔIL-1β_8h/0_, ΔIL-6_8h/0_, and ΔTNFα _8h/0_ was evaluated to identify factors associated with initial chemokine up- and down-regulation. Subsequently, from among factors associated with chemokine dynamics in univariate analysis, independent predictors were selected in multivariate linear regression. Results obtained for each chemokine are described below and summarized in Table 2 (univariate analysis) and 3 (multivariate analysis). Patient BMI and total number of resected lymph nodes, representing the extent of surgery, were not associated with an initial change in any chemokine evaluated.

We also investigated the effect of change in leukocyte counts on chemokine dynamics. As data for leukocytes were available preoperatively and 24 h post-incision, chemokine absolute and relative levels at 24 h were evaluated. The counts of WBC and NEU increased, while those of LYM dropped following surgery (*p* < 0.001 for all). Surgery type had no effect on a change: *p* = 0.849 for WBC, *p* = 0.577 for NEU, and *p* = 0.793 for LYM. There was no correlation between chemokine levels and leukocyte counts when either absolute concentrations or chemokine-related changes (Δ_24h/0_) were analyzed 24 h post-incision, with the exception of a positive correlation between ΔEOX1_24h/0_ and ΔLYM_24h/0_ (*r* = 0.28, *p* = 0.042).

#### 3.3.1. Interleukin-8 (IL-8)

ΔIL-8_8h/0_ positively correlated with LoS, EBL, ΔIL-1β_8h/0_, ΔTNFα_8h/0_, and ΔIL-6_8h/0_. The correlations with ΔIL-1β_8h/0_ and ΔTNFα_8h/0_ were significant exclusively in OCS and the correlation with LoS in RACS (Table 2). In multivariate analysis, LoS, EBL, ΔTNFα_8h/0_, and ΔIL-6_8h/0_ were independent predictors of ΔIL-8_8h/0_, explaining 62% in its variability. With excluded EBL, surgical approach was retained as an independent predictor instead, and the model explained 68% of ΔIL-8_8h/0_ variability (Table 3).

#### 3.3.2. Monocyte Chemoattractant Protein 1 (MCP-1)

ΔMCP-1_8h/0_ positively correlated with age, CCS, EBL, ΔIL-1β_8h/0_, ΔTNFα_8h/0_, and ΔIL-6_8h/0_. The correlations with age, ΔIL-1β_8h/0_, and ΔTNFα_8h/0_ were significant exclusively in OCS (Table 2). In multivariate analysis, only ΔIL-6_8h/0_ was retained in the model, explaining 62% of ΔMCP-1_8h/0_ variability (Table 3). 

#### 3.3.3. Macrophage Inflammatory Protein 1α (MIP-1α)

ΔMIP-1α_8h/0_ positively correlated with ΔIL-1β_8h/0_ and ΔTNFα_8h/0_, regardless of surgical approach (Table 2). Of these, ΔIL-1β_8h/0_ was independently associated with ΔMIP-1α_8h/0_, explaining 26% in its variability (Table 3). ASA was not included in the analysis, as its relation with ΔMIP-1α_8h/0_ was non-linear (see Supplementary Figure S6).

#### 3.3.4. Macrophage Inflammatory Protein 1β (MIP-1β)

ΔMIP-1β_8h/0_ positively correlated with ΔIL-1β_8h/0_ and ΔTNFα_8h/0_ exclusively in RACS (Table 2). In this group, in multivariate analysis, ΔIL-1β_8h/0_ was an independent predictor of ΔMIP-1β_8h/0_, explaining 16.5% in its variability (Table 3).

#### 3.3.5. Eotaxin 1 (EOX1/CCL11)

ΔEOX1_8h/0_ inversely correlated with length of surgery in OCS and positively with ΔIL-1β_8h/0_, ΔTNFα_8h/0_, and ΔIL-6_8h/0_. ΔEOX1_8h/0_ in RACS correlated only with ΔIL-1β_8h/0_ and ΔTNFα_8h/0_, but the association was stronger (Table 2). In multivariate analysis, ΔTNFα _8h/0_ was an independent predictor of ΔEOX1_8h/0_, explaining 38% in its variability (Table 3).

#### 3.3.6. Regulation on Activation, Normal T Cell Expression and Secretion (RANTES/CCL5)

ΔRANTES_8h/0_ positively correlated with ΔIL-1β_8h/0_ and ΔTNFα_8h/0_ in RACS (Table 2). In multivariate analysis, ΔTNFα_8h/0_ was an independent predictor of ΔRANTES_8h/0_, explaining 17.5% of its variability if analyzed in a whole cohort, and 31% if analyzed in RACS (Table 3).

### 3.4. Chemokines and Clinical Outcomes

#### 3.4.1. Surgical Site Infections (SSIs)

**Figure 7:**
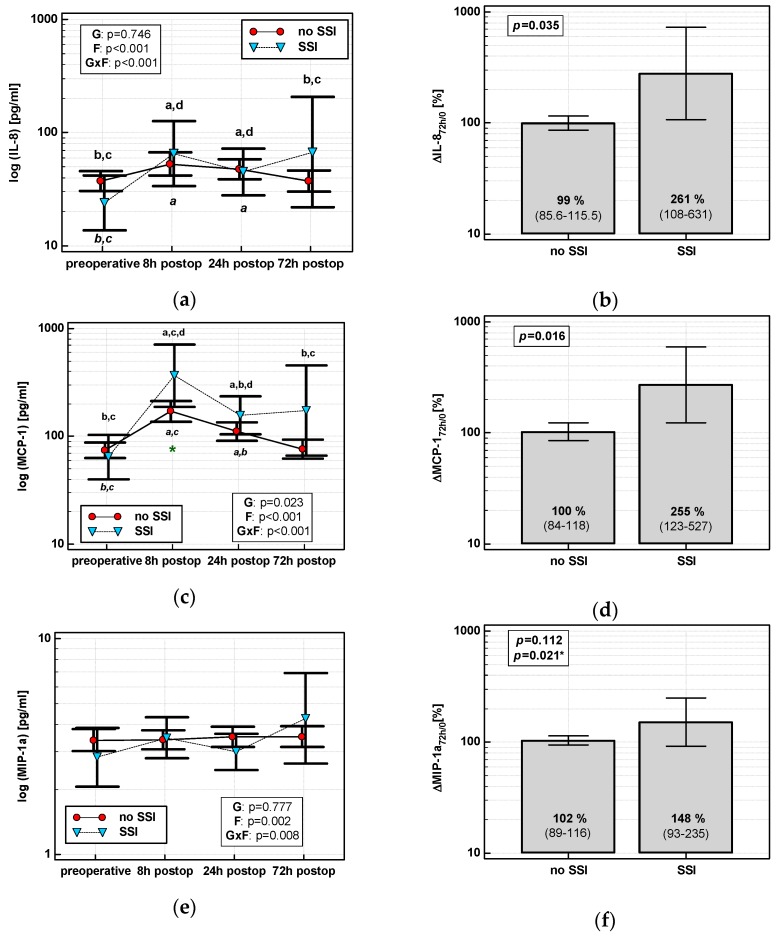

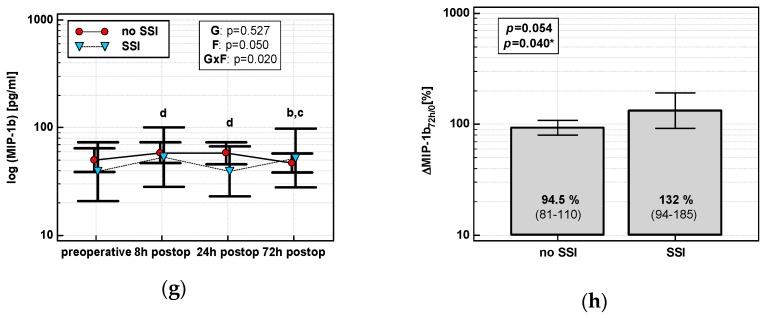
Chemokine dynamics and surgical site infections (SSIs): (**a**) IL-8 time course; (**b**) ΔIL-8_72h/0_; (**c**) MCP-1 time course; (**d**) ΔMCP-1_72h/0_; (**e**) MIP-1α time course; (**f**) ΔMIP-1α_72h/0_; (**g**) MIP-1β time course; and (**h**) ΔMIP-1β_72h/0_. Data are presented as geometric means with a 95% CI and analyzed using two-way repeated measures ANOVA (chemokine time course) with statistical significance of group (G), factor (F; time), and their interaction (G × F) effects given in the figure insert or using a *t*-test for independent samples. *P* value with asterisks denotes statistical significance after adjustment to differences in surgery type. Statistically significant differences between particular time points with no SSI (above marker, straight script) and with an SSI (below marker, italics) are marked by lower script letters with “a” denoting preoperative measurement, “b” denoting 8 h, “c” denoting 24 h, and “d” denoting 72 h. Significant difference between groups at a given time point is denoted by a green asterisk.

The time course of IL-8, MCP-1, and both MIP chemokines was significantly altered in patients who subsequently developed an SSI (interaction *p* < 0.05 for all) (Figure 7a,c,e,g). Analysis of percentage changes at 72 h showed that ΔIL-8_72h/0_ and ΔMCP-1_72h/0_ were significantly higher in patients developing SSIs (Figure 7b,d). ΔMIP-1α_72h/0_ and ΔMIP-1β_72h/0_ association gained significance after accounting for surgery type (Figure 7f,h). The remaining chemokines did not differ significantly with respect to SSIs. The analysis of data at 72 h to assess the predictive power of SSIs was chosen, as it was the last blood sampling for chemokine evaluation during the follow-up and it still preceded the clinical manifestation of SSI.

**Figure 8:**
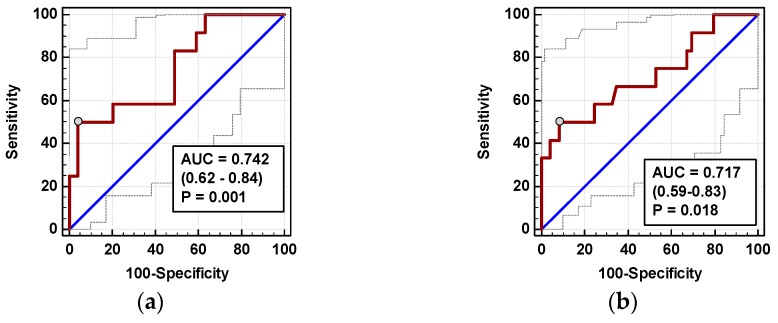
Chemokines as SSI predictors: (**a**) ΔIL-8_72h/0_ and (**b**) ΔMCP-1_72h/0_. Data are presented as receiver operating characteristics (ROC) curves (solid red line) with a 95% CI (dashed lines) of a potential marker as compared to a chance marker (diagonal blue line). Accuracy of the evaluated chemokine is shown as the area under the ROC curve (AUC) with a 95% CI and significance of chemokine AUC being different from a chance marker (AUC = 0.5).

Individually, both percentage changes of IL-8 and MCP-1 at 72 h (ΔIL-8_72h/0_ and ΔMCP-1_72h/0_, respectively) displayed a fair accuracy as prognosticators of SSI (Figure 8).

In logistic regression, out of clinical variables reportedly associated with an increased risk of SSI and chemokine dynamic-derived measures significantly associated with SSI in univariate analysis, an open procedure and ΔMCP-1_72h/0_ were independently associated with SSI, displaying 82% accuracy, 92% sensitivity, and 70% specificity in predicting SSIs (Table 4).

#### 3.4.2. Anastomotic Leak (AL)

**Figure 9:**
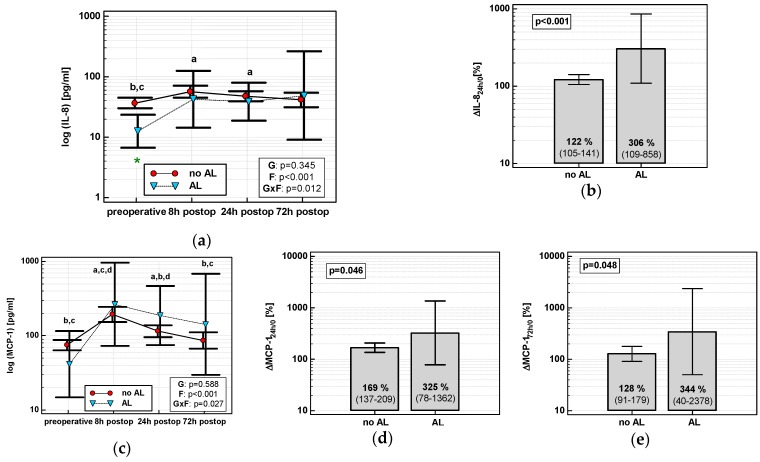
Chemokine dynamics and anastomotic leak (AL): (**a**) IL-8 time course; (**b**) ΔIL-8_24h/0_; (**c**) MCP-1 time course; (**d**) ΔMCP-1_24h/0_; and (**e**) ΔMCP-1_72h/0_**.** Data are presented as geometric means with a 95% CI, and analyzed using two-way repeated measures ANOVA (chemokine time course) with statistical significance of group (G), factor (F; time), and their interaction (G × F) effects given in the figure insert or using a *t*-test for independent samples. Statistically significant differences between particular time points within no AL (above marker, straight script) and with AL (below marker, italics) are marked by lower script letters with “a” denoting preoperative measurement, “b” denoting 8 h, “c” denoting 24 h, and “d” denoting 72 h.

The time course of IL-8 and MCP-1 was significantly altered in patients who subsequently developed AL (interaction *p* < 0.05 for both) (Figure 9a,c). AL occurred in four OCS patients. In OCS group, ΔIL-824h/0, ΔMCP-124h/0, and ΔMCP-172h/0 were significantly higher in patients developing AL (Figure 9b,d,e).

**Figure 10:**
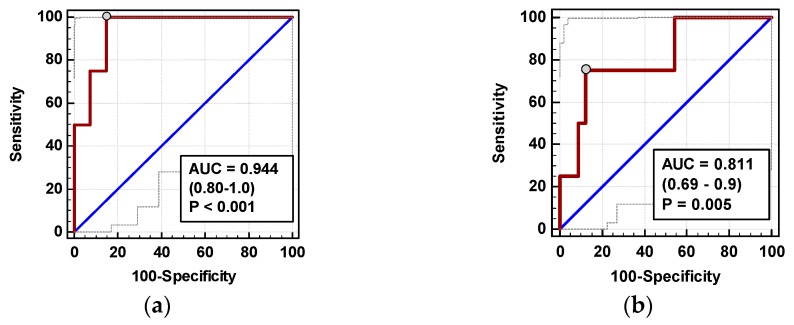
Chemokines as AL predictors: (**a**) ΔIL-8_72h/0_ and (**b**) ΔMCP-1_72h/0_. Data are presented as receiver operating characteristics (ROC) curves (solid red line) with a 95% CI (dashed lines) of a potential marker as compared to a chance marker (diagonal blue line). Accuracy of the evaluated chemokine is shown as the area under the ROC curve (AUC) with a 95% CI and significance of chemokine AUC being different from a chance marker (AUC = 0.5).

In multivariate analysis, out of clinical variables reportedly associated with increased risk of AL and relevant chemokine dynamic-derived measures, ΔIL-8_24h/0_ was an independent predictor of AL (Table 4). Its 94% accuracy was superior to ΔMCP-1_72h/0_ (Figure 10), whereas that of ΔMCP-1_24h/0_ was insignificant (*p* = 0.115).

#### 3.4.3. Restoration of Bowel Function (RoBF)

**Figure 11:**
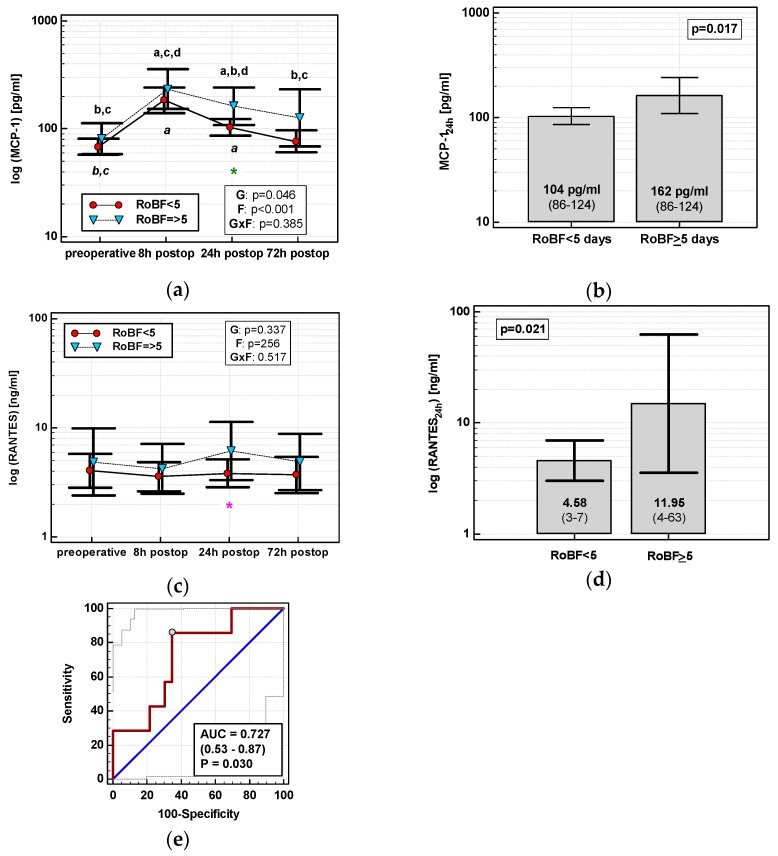
Chemokines and restoration of bowel function (RoBF): (**a**) MCP-2 time course; (**b**) MCP-1_24h_ in patients with RoBF < and ≥ 5 days; (**c**) RANTES time course; (**d**) RANTES_24h_ in patients with RoBF < and ≥ 5 days; and (**e**) MCP-1_24h_ as a predictor of RoBF ≥ 5 days in RACS. Data are presented as geometric means with a 95% CI and analyzed using two-way repeated measures ANOVA (chemokine time course) with statistical significance of group (G), factor (F; time), and their interaction (G × F) effects given in the figure insert or using a *t*-test for independent samples (mean comparisons). Statistically significant differences between particular time points within RoBF < 5 (above marker, straight script) and RoBF ≥ 5 (below marker, italics) are marked by lower script letters with “a” denoting preoperative measurement, “b” denoting 8 h, “c” denoting 24 h, and “d” denoting 72 h. Significant difference between groups at a given time point is denoted by a green asterisk (for whole cohort) or pink asterisk (if valid only in RACS). Data in the (**e**) panel are presented as receiver operating characteristics (ROC) curves (solid red line) with a 95% CI (dashed lines) of a potential marker as compared to a chance marker (diagonal blue line). Accuracy of the evaluated chemokine is shown as the area under the ROC curve (AUC) with a 95% CI and significance of chemokine AUC being different from a chance marker (AUC = 0.5).

None of the time courses of the evaluated chemokines were significantly altered by the time of RoBF. However, the absolute concentrations of MCP-1 and RANTES differed with respect to RoBF (Figure 11a,c). MCP-1_24h_ was significantly higher in patients with RoBF ≥ 5 days (cut-off selected based on median) (Figure 11b). RANTES_24h_ was also higher in patients with RoBF ≥ 5 days, but exclusively in RACS (Figure 11d). As a RoBF ≥ 5 marker, MCP-1_24h_ was significantly better than a chance marker exclusively in RACS, displaying 73% accuracy (Figure 11e), but the accuracy of RANTES_24h_ was insignificant (*p* = 0.110).

In multivariate analysis, out of clinical variables reportedly associated with an increased risk of prolonged postoperative ileus and relevant chemokine data, dichotomized length of surgery and MCP-1 concentration at 24 h were selected as independent predictors of RoBF ≥ 5 days, displaying 73% accuracy, 100% sensitivity, and 43% specificity (Table 4).

#### 3.4.4. Length of Hospital Stay (LoHS)

**Figure 12:**
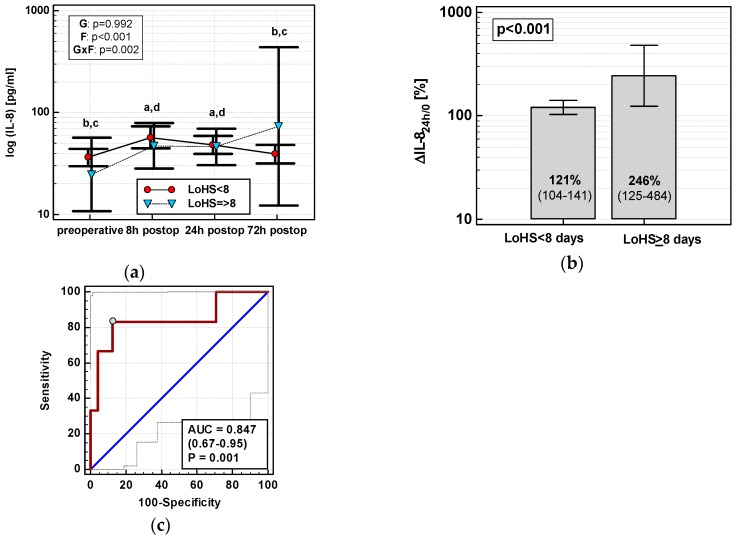
IL-8 and length of hospital stay (LoHS) in OCS: (**a**) IL-8 time course; (**b**) ΔIL-8_24h/0_ in patients with LoHS < and ≥ 8 days; and (**c**) ΔIL-8_24h/0_ as a predictor of LoHS ≥ 8 days. Data are presented as geometric means with a 95% CI and analyzed using two-way repeated measures ANOVA (chemokine time course) with statistical significance of group (G), factor (F; time), and their interaction (G × F) effects given in the figure insert or using a *t*-test for independent samples (mean comparisons). Statistically significant differences between particular time points within LoHS < 8 days (above marker, straight script) are marked by lower script letters with “a” denoting preoperative measurement, “b” denoting 8 h, “c” denoting 24 h, and “d” denoting 72 h. (**c**) Panel data are presented as receiver operating characteristics (ROC) curves (solid red line) with a 95% CI (dashed lines) of a potential marker as compared to a chance marker (diagonal blue line). Accuracy of the evaluated chemokine is shown as the area under the ROC curve (AUC) with a 95% CI and significance of chemokine AUC being different from a chance marker (AUC = 0.5).

Exclusively in OCS, ΔIL-8_24h/0_ (r = 0.55, *p* = 0.002), MCP-1_24h_ (r = 0.40, *p* = 0.028), ΔMCP-1_24h/0_ (r = 0.38, *p* = 0.039), ΔMIP-1α_24h/0_ (r = 0.38, *p* = 0.040), and RANTES_24h_ (r = 0.50, *p* = 0.005) positively correlated with LoHS. In OCS, only the IL-8 time course differed with respect to LoHS when the parameter was dichotomized (< or ≥ 8 days, based on mean LoHS in OCS) (Figure 12a). Of dynamics-derived measures, only ΔIL-8_24h/0_ was significantly associated with dichotomized LoHS (Figure 12b), and its accuracy as a LoHS ≥ 8 marker was good (Figure 12c).

In multivariate analysis, out of clinical variables reportedly associated with increased risk for prolonged hospital stay and relevant chemokine data, an open surgery, tumor location in the rectum, and RANTES_24h_ were independently associated with LoHS in a whole cohort, explaining 26% of its variability. If restricted to OCS patients, ΔIL-8_24h/0_ and RANTES_24h_ were independently associated with LoHS (Table 4).

## 4. Discussion

Robot-assisted surgery has the short-term clinical advantages of a laparoscopic approach, namely decreased postoperative pain, lower rate of complications, and quicker restoration of bowel function reflected in shorter hospitalization [8]. However, robotic procedures require longer operating times on average and render patients prone to positioning- and prolonged-anesthesia-related complications [9]. These adverse effects may diminish the benefits of minimized surgical invasiveness. As such, results from studies unraveling the biochemical contexts of laparoscopy cannot be simply carried over to robotic surgery. Taking into account the increasing popularity of robotic technology, the paucity of data concerning the impacts of robot-assisted colorectal surgery on the body’s response to trauma is surprising. Shibata et al. [10] were the first to compare open, laparoscopic, and robotic approaches, but focused their study on monocyte HLA-DR expression, lymphocyte subtype distribution, and C-reactive protein. Subsequently, our group demonstrated attenuated inflammatory response following RACS with diminished IL-6, and procalcitonin elevation [6] and beneficial dynamics of IL-7 [6]. However, to the best of our knowledge, the issue of chemokine response following robotic and open colorectal surgery has not been previously addressed.

Here, we examined the temporal changes in IL-8, MCP-1, MIP-1α and β, eotaxin-1, and RANTES, key chemokines orchestrating leukocyte migration. While IL-8 is a key chemoattractant for neutrophils and eotaxin-1 for eosinophils, the functionality of MCP-1, MIPs, and RANTES largely overlap, as all those chemokines recruit monocytes/macrophages, T lymphocytes, natural killer cells (NK), and immature dendritic cells (DC) [11]. Surgical approach had no effect on the dynamics of eotaxin-1 and subtly altered the dynamics of MIPs. However, RACS clearly attenuated the up-regulation of IL-8 and MCP-1 and was associated with a drop in RANTES. Findings on reduced inflammatory response also in terms of chemokines corroborate reports of other authors [12,13], although not all [14], on various abdominal MIS. Less accented chemokine up-regulation might be beneficial, as excessive neutrophil and/or monocyte infiltration contributes to postoperative pulmonary complications [15] and postoperative ileus [16,17].

To provide mechanistic insights explaining chemokine dynamics, we evaluated the effect of patient-related and surgery-related clinical data other than surgical approach. Among patient-related data, we expressed age, sex, BMI, preoperative anemia, and general health condition, in terms of two scores: the American Society of Anesthesiologists (ASA) physical status classification, and Charlson Comorbidity Score (CCS). Among surgery-related parameters, surgical procedure (abdominoperineal resection, low anterior resection, right hemicolectomy, left hemicolectomy, and sigmoid resection), extent of surgical trauma expressed in terms of number of harvested lymph nodes, magnitude of blood loss and transfusions, and surgery length were assessed. Of those, the initial chemokine change was affected only by age (IL-8 and MCP-1), length of surgery (IL-8, MCP-1, and eotaxin), and estimated blood loss (IL-8, MCP-1). Additionally, patients’ health condition affected MCP-1 and RANTES as shown by the positive correlation between an initial increase in MCP-1 and CCS and by an inverse association between initial change in RANTES and preoperative anemia. Surgical procedure, associated with tumor location, significantly affected an initial rise in IL-8 and MIP-1α, but this observation resulted from distinct chemokine dynamics in only two patients undergoing abdominoperineal resection and as such lacks credibility. Regardless, sole length of surgery and estimated blood loss, exclusively for IL-8, were found to contribute significantly when co-analyzed with other variables and proinflammatory cytokines. Interestingly, patients’ age, previously demonstrated to correlate with a prolonged hospital stay and a higher potential for complications and mortality following colorectal surgery [18], was significant only in a univariate analysis and exclusively if dichotomized. The length of surgery among our RACS patients on average was significantly longer than observed in the OCS group, which may have resulted from the extended preparation and set-up time of the surgical robot and, in our case, from the fact of our surgeons being still on the learning curve. Of note, early publications on the learning curve in robotic colorectal surgery have suggested that robotic surgeons become efficient after only 15–25 robotic procedures [19]. In contrast, more recent papers have shown a much longer learning process that may require over 100 procedures [20]. Taken together, those findings imply that chemokine elevation may be further reduced by shortening the operating time.

Contrary to relatively limited impact on initial chemokine response, more clinical variables and more substantially affected chemokine dynamics during further observation. Here, we demonstrated that normal weight was associated with attenuation, and transfusions with accelerated inflammatory response in terms of MCP-1, MIP-1β, and RANTES dynamics. Correspondingly, chronic obesity is considered a state of a low-grade inflammation and tightly linked with an immune dysregulation which, in patients undergoing colorectal surgery, translates into an increased risk of complications, including anastomotic leak and sepsis [21]. Furthermore, perioperative transfusions in patients undergoing colorectal surgery are associated with adverse clinical outcomes, such as decreased survival, increased postoperative infections, anastomotic complications, and others, although whether the effect is mediated by suppressed immune function or not is unresolved [22]. 

Leukocytes are an important source of chemokines, the expression/secretion of which are regulated by TNFα, IL-1β, and IL-6 [23,24,25,26]. Therefore, change in leukocyte count and the dynamic in key proinflammatory cytokines were evaluated as potential triggers of chemokine up-regulation. Although WBC and NEU counts increased on the first day, similar to chemokines, there was no direct correlation between their absolute or relative levels, implying that other cells (e.g., endothelial cells or fibroblasts) are likely to contribute to chemokine up-regulation. As expected, chemokines positively correlated with proinflammatory cytokines. In the cases of MCP-1 and IL-8, these associations were stronger with IL-6, probably due to the elevation of IL-6 being secondary to IL-1β and TNFα response and/or longer half-life of IL-6. Also, these correlations were exclusively present, or stronger, in OCS, which might result from wider dynamic range of cytokine/chemokine concentrations. IL-6 up-regulation was the additional (IL-8) or sole (MCP-1) independent predictor of initial chemokine rise, implying that its diminished elevation in RACS [6] mediates attenuated MCP-1 and IL-8 response following RACS. Of potential clinical and inflammatory triggers evaluated here, MIPs-1’s dynamics were dependent solely on IL-1β, and RANTES and eotaxin-1 on that of TNFα. In fact, this close relationship between chemokines and proinflammatory cytokines, primary stress responders, is likely explain the loss of significance of clinical variables affecting initial chemokine elevation in multivariate analysis when assessed together with IL-1β, TNFα, and IL-6. 

To determine the clinical relevance of differences in chemokine dynamics, the association of their absolute concentrations and percentage changes with adverse clinical events was evaluated. We focused on those previously found to be improved with the robotic approach [8,27], namely, wound infection, anastomotic leak, postoperative ileus, and prolonged hospitalization. Since the occurrence of these events did not manifest until the third postoperative day, the potential of chemokines (at 24 and 72 h) as predictive markers was examined as well. In cases of postoperative ileus, the earliest RoBF was recorded on the second day; therefore, exclusively chemokines at 24 h were analyzed. It is worth noticing that relative changes in chemokines, rather than their absolute concentrations, were significantly associated with various clinical outcomes. Unlike absolute concentrations, which are dependent on the initial chemokine level and thus affected by cancer stage and location [28,29], relative values seem to better reflect chemokine response to analyzed triggers/events. 

In line with chemokine primary function (that is, leukocyte trafficking towards site of injury [23]), and corroborating previous findings [30], we showed up-regulation of all chemokines at 72 h to be associated with SSIs. Of these, only MCP-1 elevation and OCS were independent predictors of SSIs with a combined accuracy of 83% as predictive SSI markers, even following adjustment to known SSI risk factors such as prolonged operating time, ASA ≥ 3, perioperative blood transfusions, older age, cancer dissemination, and male sex [31,32]. Additionally, MCP-1 and IL-8 were more up-regulated in patients whose anastomosis leaked, and percentage increase in IL-8 at 24 h was an independent AL predictor, displaying 94% accuracy. Importantly, IL-8 remained associated with AL despite including clinical variables in the analysis that are known to be AL risk factors in colorectal surgery, namely, male sex, older age, obesity, longer operation time, blood transfusions, ASA ≥ 3, and cancer dissemination [33,34]. Although promising and corroborating our earlier observation in rectal cancer [35], this result has to be confirmed on a larger population. The reported incidence of AL, one of the most dreaded complications, ranges from 1 to 20%, but strongly depends on the anatomic location of the anastomosis with colocolonic leak rates as low as 0–2% [36]. Since AL occurred exclusively in OCS, our analysis was limited to these patients in order to not falsely lower chemokine levels in the non-AL group by including RACS patients, and, consequently, it was even more underpowered. The up-regulation of IL-8, MCP-1, and MIP-1α secretion was also associated with prolonged hospitalization, with IL-8 elevation at 24 h being an independent predictor of LoHS ≥ 8 days. The association was significant exclusively in OCS, probably due to a wider range of LoHS, but did not translate into IL-8 being a good predictive marker of LoHS. In multiple regression with LoH as the continuous dependent variable, percentage change in IL-8 together with absolute concentration of RANTES at 24 h post-incision were its independent predictors among OCD patients even after adjustment for known risk factors for prolonged hospitalization, such as older age, comorbidities, and tumor subsite [37]. Yet, when analyzed in a whole cohort, open surgery (also a risk factor [37]) and rectal location of the primary tumor in addition to RANTES found independent predictors of prolonged hospitalization. 

Excessive monocyte/macrophage infiltration contributes to late phase of postoperative ileus, a phenomenon that may delay hospital discharge and add to postoperative morbidity. Minimizing invasiveness of intervention and avoiding major inflammatory events are believed to yield the greatest benefit in managing postoperative ileus [16,17,38]. Here, we found MCP-1, a key chemoattractant for monocytes/macrophages, to be significantly higher in patients with delayed RoBF. As a marker of delayed RoBF in RACS patients, MCP-1 was characterized by 73% accuracy. Our findings agree well with observations on increased MCP-1 bowel expression being triggered by colonic manipulation and proportional to the extent of surgery [16,17,39]. MCP-1 also remained significantly associated with delayed RoBF following adjustment to potential risk factors of prolonged postoperative ileus such as male gender, older age, prolonged operation time, cancer dissemination, blood transfusions, ileostomy, AL, extent of surgery, obesity, and ASA ≥ 3 [40], of which only the length of surgery, dichotomized, was found significant. Delayed RoBF was also preceded by elevated RANTES. The RANTES association with postoperative ileus might be additionally mediated by mast cells. Mast cells are key cellular players in allergies, but are also involved in processes relevant in gastrointestinal surgery, namely, wound healing and postoperative ileus [41], and RANTES is among the chemokines that participate in their trafficking [42].

Surgery-induced immune imbalance renders cancer patients susceptible to accelerated growth of residual cancer cells [2,43]. As such, attenuated inflammatory response in terms of proangiogenic [23] IL-8, reported here, may prove advantageous for RACS patients. Since MCP-1 promotes unfavorable Th2 response [44], its diminished up-regulation might benefit RACS patients in terms of improved Th1/Th2 balance. Cancer disease per se is associated with down-regulated Th1 response, enabling tumors to escape immune surveillance, and surgery deepens the imbalance even further [45]. Although the dynamics of MIPs-1 were subtle, their up-regulation at 24 h as compared with 8 h in RACS may contribute to a more favorable shift in Th1/Th2 balance as well, as both chemokines are associated with Th1 responses [46]. However, in line with the crucial role attributed to MIPs-1 in antimicrobial immune responses [46,47], the dynamics of both chemokines at 72 h were dominated by infection, and MIP-1α and β were significantly elevated in patients who subsequently developed SSI.

Although evidence seems to favor the application of robotics in rectal over colon cancer surgery, there was an unintentional prevalence of patients with colonic cancer in our RACS group. However, robotic colon surgery is steadily gaining popularity, having certain advantages over laparoscopy. In fact, even in the field of rectal surgery, solid evidence supporting use of robotics is lacking. In general, the application of robotics in colorectal surgery has been a subject of passionate debate among surgical societies for the past decade, and one which has not reached any final conclusion. Moreover, recent reports suggest that the number of robotic colectomies rose from 3.7 to 17.1% of all minimally invasive segmental colon resections [48]. Furthermore, as reported by another large registry study, robotic right colectomy was associated with increased operative time, but lowered conversion rates and shortened length of hospitalization when compared to laparoscopy [49]. 

## 5. Conclusions

Chemokine up-regulation is attenuated following RACS and associated with clinical variables directly linked with the type of surgical approach such as magnitude of blood loss and/or severity of inflammatory response, as well as patient-related parameters such as excessive weight, older age, or anemia prior to surgery. Chemokine dynamics appear to be beneficial in RACS due to a quicker restoration of bowel function and favored Th1 immune response. Its monitoring may prove useful in predicting SSI and AL.

## Figures and Tables

**Table 1:** Characteristics of study population.

Parameter	Open Surgery	Robotic Surgery	*p* Value
Size, *n*	31	30	-
Patient-related:			
Sex distribution (F/M)	14/17	7/23	0.127
Age (years), median (95% CI)	68 (65–76)	67 (61.5–71.8)	0.302
Patients ≥ 75 years, *n*	12/19	8/22	0.416
BMI ^1^ (kg/m ^2^), mean (95% CI)	26.8 (25.1–28.5)	26.6 (24.8–28.4)	0.852
Overweight/obese patients ^1^, *n*	10/20	13/16	0.430
ASA (1/2/3), *n*	6/20/5	5/20/5	0.830
Charlson Comorbidity Score, median (95% CI)	5 (4–5)	4.5 (4–5.8)	0.988
HGB (g/dL), mean (95% CI)	11.9 (11.2–12.6)	12.3 (11.6–13)	0.456
Anemia ^2^ (no/yes), *n*	12/19	9/21	0.592
WBC (×10 ^3^/mm ^3^), mean (95% CI)	7.01 (6.23–7.78)	7.26 (6.47–8.04)	0.645
NEU (×10 ^3^/mm ^3^), mean (95% CI)	4.72 (4.1–5.34)	4.9 (4.3–5.5)	0.679
LYM (×10 ^3^/mm ^3^), mean (95% CI)	1.5 (1.32–1.68)	1.45 (1.19–1.72)	0.758
Cancer-related:			
Stage distribution (0/I/II/III/IV), *n*	2/2/15/9/3	2/3/11/12/2	0.839
Stage T distribution (Tis/T1/T2/T3/T4), *n*	2/1/1/20/7	2/0/5/16/7	0.393
Stage N distribution (N0/N1/N2), *n*	19/4/8	16/8/6	0.395
Stage M distribution (M0/M1), *n*	28/3	28/2	0.970
Grade (G1/G2/G3/G4), *n*	3/21/4/1	5/18/5/0	0.519
Tumor location (LC/RC/RE), *n*	11/6/14	7/12/11	0.199
Surgery-related:			
Surgical procedure (APR/LAR/RH/LH/SR), *n*	1/13/6/1/10	1/10/12/3/4	0.203
Length of surgery (min), median (95% CI)	125 (115–150)	205 (191–240)	<0.001
Length of surgery ^3^ (≤165 min/>165 min), *n*	26/5	6/24	<0.001
Total nodes resected, mean (95% CI)	15.7 (13.5–17.9)	14.8 (12.4–17.2)	0.568
Total nodes resected ^3^ (<14/≥14), *n*	10/21	16/14	0.124
Estimated blood loss (mL), median (95% CI)	200 (150–200)	50 (50–100)	<0.0001
Estimated blood loss ^3^ (≤100 mL/>100 mL), *n*	6/25	25/6	<0.001
Transfusions, *n* (%)	5 (16.1%)	2 (6.7%)	0.425
Frequency of stomas, *n* (%)	5 (16.1%)	5 (16.7%)	1.0
Restoration of bowel function (<5/≥5 day), *n*	19/12	23/7	0.270
Length of hospital stay, days (range)	7.6 (4–20)	5.8 (4–8)	0.020
Clavien-Dindo score (≤2/3/4/5), *n*	26/3/2/0	29/1/0/0	0.207
Complications ^4^, *n* (%):	4 (12.9%)	0	0.113
Anastomotic leak (AL), *n*	27/4	30/0	0.113
Surgical site infection, *n* (%)	10 (32.3%)	2 (6.7%)	0.013
Superficial	3	2	
Deep	3	0	
Organ-space	4	0	

*n*, number of patients; F/M, female-to-male ratio; CI, confidence interval; BMI, body mass index; ASA, physical status classification system; HGB, hemoglobin; WBC, white blood cell count; NEU, neutrophil count; LYM; lymphocyte count; LC, left colon; RC, right colon; RE, rectum; APR, abdominoperineal resection; LAR, low anterior resection; RH, right hemicolectomy; LH; left hemicolectomy; SR, sigmoid resection; ^1^, available for 59 patients; ^2^, defined as HGB < 12 g/dL in women and <13.5 g/dL in men; ^3^, dichotomized based on median in whole population; ^4^, surgical complications with Clavien-Dindo score ≥ 3.

**Table 2:** Possible predictors of initial chemokine rise: univariate analysis.

Variables	Wiek	CCS	LoS	EBL	ΔIL-1β_8h/0_	ΔTNFα_8h/0_	ΔIL-6_8h/0_
ΔIL-8_8h/0_	ns	ns	r = 0.45, *p* = 0.012^R^	r = 0.38, *p* = 0.002	r = 0.63, *p* < 0.001^O^	r = 0.54, *p* = 0.002^O^	r = 0.70, *p* < 0.001^O^ r = 0.58, *p* < 0.001^R^
ΔMCP-1_8h/0_	r = 0.38, *p* = 0.035^O^	r = 0.28, *p* = 0.028	ns	r = 0.35, *p* = 0.006	r = 0.40, *p* = 0.024^O^	r = 0.40, *p* = 0.025^O^	r = 0.75, *p* < 0.001^O^ r = 0.79, *p* < 0.001^R^
ΔMIP-1α_8h/0_	ns	ns	ns	ns	r = 0.54, *p* = 0.002^O^ r = 0.51, *p* = 0.002^R^	r = 0.49, *p* = 0.005^O^ r = 0.49, *p* = 0.006^R^	ns
ΔMIP-1β_8h/0_	ns	ns	ns	ns	r = 0.41, *p* = 0.026^R^	r = 0.39, *p* = 0.034^R^	ns
ΔEOX_8h/0_	ns	ns	r = −0.37, *p* = 0.043^O^	ns	r = 0.36, *p* = 0.045^O^ r = 0.67, *p* < 0.001^R^	r = 0.44, *p* = 0.014^O^ r = 0.73, *p* < 0.001^R^	r = 0.37, *p* = 0.042^O^
ΔRANTES_8h/0_	ns	ns	ns	ns	r = 0.42, *p* = 0.020^R^	r = 0.55, *p* = 0.002^R^	ns

CCS, Charlson Comorbidity Score; LoS, length of surgery; EBL, estimated blood loss; ^O^, relevant in open surgery group; ^R^, relevant in robotic group; ns, non-significant.

**Table 3:** Possible predictors of initial chemokine rise: multiple linear regression.

Dependent Variable	Entered Explanatory Variables	Retained Variables Coeff. b; r_p_, Significance	Goodness of Fit Constant; R^2^; F-Ratio, Significance
ΔIL-8_8h/0_	surgery (OCS as 1), age^1^, LoS, EBL, APR^2^, ΔIL-1β_8h/0_, ΔTNFα_8h/0_, ΔIL-6_8h/0_	LoS: b = 0.001; r_p_ = 0.43, *p* < 0.001 EBL: b = 0.001; r_p_ = 0.45, *p* < 0.001 ΔTNFα_8h/0_: b = 0.397; r_p_ = 0.38, *p* = 0.003 ΔIL-6_8h/0_: b = 0.238; r_p_ = 0.61, *p* < 0.0001	0.251; R^2^ = 0.623; F = 23.12, *p* < 0.0001; with surgery instead of EBL: 0.294; R^2^ = 0.584; F = 19.66, *p* < 0.0001
ΔMCP-1_8h/0_	surgery, CCS, age^1^, LoS^1^, EBL, ΔIL-1β_8h/0_, ΔTNFα_8h/0_, ΔIL-6_8h/0_	ΔIL-6_8h/0_: b = 0.472; r_p_ = 0.79, *p* < 0.0001	0.942; R^2^ = 0.621; F = 96.65, *p* < 0.0001
ΔMIP-1α_8h/0_	APR^2^, ΔIL-1β_8h/0_ ΔTNFα_8h/0_	ΔIL-1β_8h/0_: b = 0.359; r_p_ = 0.51, *p* < 0.0001	1.279; R^2^ = 0.263; F = 21, *p* < 0.0001
ΔMIP-1β_8h/0_ (in RACS)	ΔIL-1β_8h/0_ ΔTNFα_8h/0_	ΔIL-1β_8h/0_: b = 0.312; r_p_ = 0.41, *p* = 0.026	1.401; R^2^ = 0.165; F = 5.54, *p* = 0.026
ΔEOX_8h/0_	LoS, ΔIL-1β_8h/0_, ΔTNFα_8h/0_, ΔIL-6_8h/0_	ΔTNFα_8h/0_: b = 0.499; r_p_ = 0.62, *p* < 0.0001	0.933; R^2^ = 0.378; F = 35.89, *p* < 0.0001
ΔRANTES_8h/0_	surgery, anemia, ΔIL-1β_8h/0_ ΔTNFα_8h/0_	ΔTNFα_8h/0_: b = 0.769; r_p_ = 0.42, *p* < 0.001 in RACS: ΔTNFα_8h/0_: b = 1.135; r_p_ = 0.55, *p* = 0.002	0.358; R^2^ = 0.175; F = 12.54, *p* < 0.001 in RACS: –0.45; R^2^ = 0.305; F = 12.3, *p* = 0.002

LoS, length of surgery; EBL, estimated blood loss; CCS, Charlson Comorbidity Score; coeff. b, regression coefficient; r_p_, partial correlation coefficient; R^2^, coefficient of determination; OCS, open colorectal surgery; RACS, robot-assisted colorectal surgery. ^1^, continuous variable dichotomized for the analysis; ^2^, dichotomized abdominoperineal resection (APR) against all other surgical procedures.

**Table 4:** Chemokines as predictors of adverse clinical events.

Logistic Regression:				
Dependent variable	Entered explanatory variables	Retained variables Coeff. b, *p*	Goodness of fit χ^2^, *p*; R_N_^2^	Accuracy AUC (95% CI), *p*; sens. and spec. (%)
SSI	surgery (OCS encoded as 1), LoS^1^, ASA^2^, BMI^1^, transfusions, age, cancer dissemination^3^, sex, ΔIL-8_72h/0_, ΔMCP-1_72h/0_, ΔMIP-1α_72h/0_, ΔMIP-1β_72h/0_	OCS: b = 1.86, *p* = 0.042 ΔMCP-1_72h/0_: b = 2.69, *p* = 0.008;	χ^2^ = 15.9, *p* < 0.001; R_N_^2^ = 0.37	0.819 (0.70–0.91), *p* < 0.0001; 92 and 70%
AL (in OCS)	sex, BMI^1^, LoS^1^, transfusions, ASA^2^, cancer dissemination^3^, age, ΔIL-8_24h/0_, ΔMCP-1_24h/0_, ΔMCP-1_72h/0_	ΔIL-8_24h/0_: b = 12, *p* = 0.037	χ^2^ = 11.2, *p* < 0.001; R_N_^2^ = 0.59	0.938 (0.78–0.99), *p* < 0.0001; 100 and 83%
RoBF	surgery, sex, age, LoS, cancer dissemination, transfusion, stomy^4^, AL, TNR, BMI^1^,ASA^2^, MCP-1_24h_, RANTES_24h_	LoS^5^: b = −1.4, *p* = 0.033 MCP-1_24_: b = 2.72, *p* = 0.017	χ^2^ = 10.4, *p* = 0.006; R_N_^2^ = 0.23	0.734 (0.60–0.84), *p* < 0.001; 100 and 43%
Multiple linear regression:				
Dependent variable	Entered explanatory variables	Retained variables Coeff. b; r_p_, *p*		Goodness of fit Constant; R^2^; F-ratio, *p*
LoHS	surgery, age, ASA^2^, subsite^6^, ΔIL-8_24h/0_, ΔMCP-1_24h/0_, MCP-1_24h_, ΔMIP-1α_24h/0_, RANTES_24h_	OCS: b = 2.01; r_p_ = 0.36, *p* = 0.007 subsite: b = 2.01; r_p_ = 0.37, *p* = 0.005 RANTES_24h_: b = 1.58; r_p_ = 0.27, *p* = 0.045		3.83; R^2^ = 0.26; F = 6.4, *p* < 0.001
LoHS (open)	age, ASA^2^, subsite^6^, ΔIL-8_24h/0_, ΔMCP-1_24h/0_, MCP-1_24h_, ΔMIP-1α_24h/0_, RANTES_24h_	ΔIL-8_24h/0_: b = 8.28, r_p_ = 0.55, *p* = 0.003 RANTES_24h_: b = 4.54; r_p_ = 0.49, *p* = 0.009		−12.4; R^2^ = 0.47; F = 11.5, *p* < 0.001

SSI, surgical site infection; AL, anastomotic leak; LoHS, length of hospital stay; RoBF, restoration of bowel function; OCS, open colorectal surgery; RACS, robot-assisted surgery; LoS, length of surgery; ASA, physical status classification system; TNR, total number of resected lymph nodes; coeff. b, regression coefficient; r_p_, partial correlation coefficient; R^2^, coefficient of determination; R_N_^2^, Nagelkerke R^2^; AUC, area under receiver operating characteristics (ROC) curve; CI, confidence interval; sens., sensitivity; spec., specificity. ^1^, analyzed either as continuous or dichotomized variable; ^2^, dichotomized as ASA < 3 and ASA ≥ 3; ^3^, analyzed either in terms of distant metastasis alone (M1 against M0) or in terms of local metastasis (N ≠ 0 vs. N0; all M1 patients were also N ≠ 0); ^4^, analyzed either as ileostomy alone or as ileostomy/colostomy; ^5^, included only when dichotomized; ^6^, dichotomized as rectal location against all others.

## Supplementary Materials

The following are available online at https://www.mdpi.com/2077-0383/8/6/879/s1. Figure S1: Effect of various clinical parameters on perioperative IL-8 dynamics; Figure S2: Effect of estimated blood loss (EBL) on percentage change in IL-8; Figure S3: Effect of various clinical parameters on perioperative MCP-1 dynamics; Figure S4: Effect of various clinical parameters on percentage change in MCP-1; Figure S5: Effect of various clinical parameters on perioperative MIP-1α dynamics; Figure S6: Effect of various clinical parameters on percentage change in MIP-1α; Figure S7; Effect of various clinical parameters on perioperative MIP-1β dynamics; Figure S8: Effect of various clinical parameters on percentage change in MIP-1β; Figure S9: Effect of various clinical parameters on perioperative eotaxin (EOX)-1 dynamics; Figure S10: Effect of physical status classification system (ASA) score on percentage change in eotaxin (EOX)-1; Figure S11: Effect of various clinical parameters on perioperative dynamics of RANTES; Figure S12: Effect of various clinical parameters on percentage change in RANTES.
