# Supplementary material for: Distinct Chemokine Dynamics in Early Postoperative Period after Open and Robotic Colorectal Surgery
Source: J Clin Med. 2019 Jun 19;8(6):879. doi: 10.3390/jcm8060879 (PMC6616914; doi:10.3390/jcm8060879)
Supplement: Supplementary file 1 [file jcm-08-00879-s001.zip › SupFig10.pdf]

Supplementary Figure S10

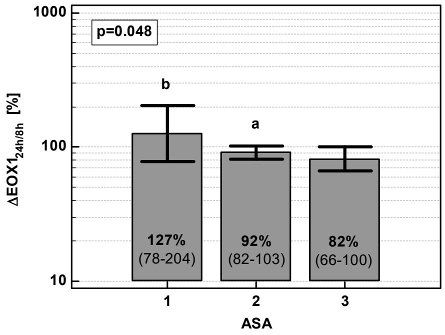

(**a**)

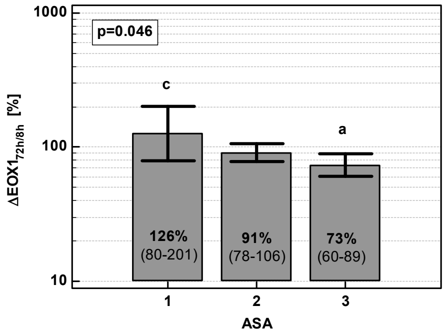

(**b**)

Supplementary Figure S10. Effect of physical status classification system (ASA) score on percentage change in eotaxin (EOX)-1: (**a**) percentage change between 24h and 8h post incision ($\Delta EOX1_{24h/8h}$); (**b**) percentage change between 72h and 8h post incision ($\Delta EOX1_{72h/8h}$). Data presented as geometric means with 95%*CI* and analyzed using one-way ANOVA. a, significantly different from ASA=1; b, significantly different from ASA=2; c, significantly different from ASA=3.
