# Supplementary material for: Distinct Chemokine Dynamics in Early Postoperative Period after Open and Robotic Colorectal Surgery
Source: J Clin Med. 2019 Jun 19;8(6):879. doi: 10.3390/jcm8060879 (PMC6616914; doi:10.3390/jcm8060879)
Supplement: Supplementary file 1 [file jcm-08-00879-s001.zip › SupFig12.pdf]

**Supplementary Figure S12**

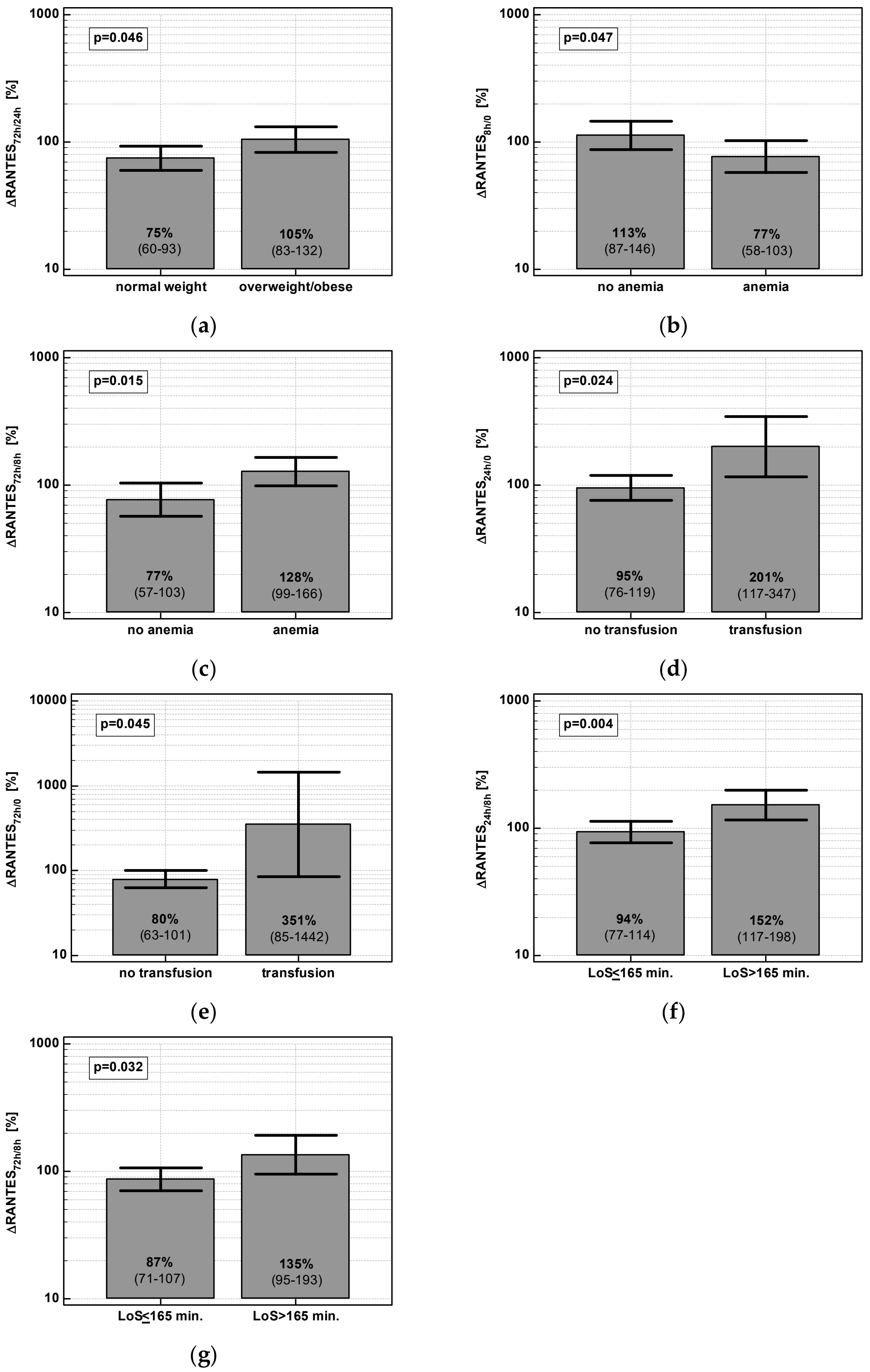


**Supplementary Figure S12.** Effect of various clinical parameters on percentage change in RANTES: (**a**) patients' BMI on $\Delta RANTES_{72h/24h}$; (**b**) anemia on $\Delta RANTES_{8h/0}$; (**c**) anemia on $\Delta RANTES_{72h/8h}$; (**d**) transfusion on $\Delta RANTES_{24h/0}$; (**e**) transfusion on $\Delta RANTES_{72h/0}$; (**f**) length of surgery (LoS) on $\Delta RANTES_{24h/8h}$; (**h**) length of surgery (LoS) on $\Delta RANTES_{72h/8h}$. Data presented as geometric means with 95%*CI* and analyzed using t-test for independent samples.
