# Supplementary material for: Distinct Chemokine Dynamics in Early Postoperative Period after Open and Robotic Colorectal Surgery
Source: J Clin Med. 2019 Jun 19;8(6):879. doi: 10.3390/jcm8060879 (PMC6616914; doi:10.3390/jcm8060879)
Supplement: Supplementary file 1 [file jcm-08-00879-s001.zip › SupFig2.pdf]

Supplementary Figure S2

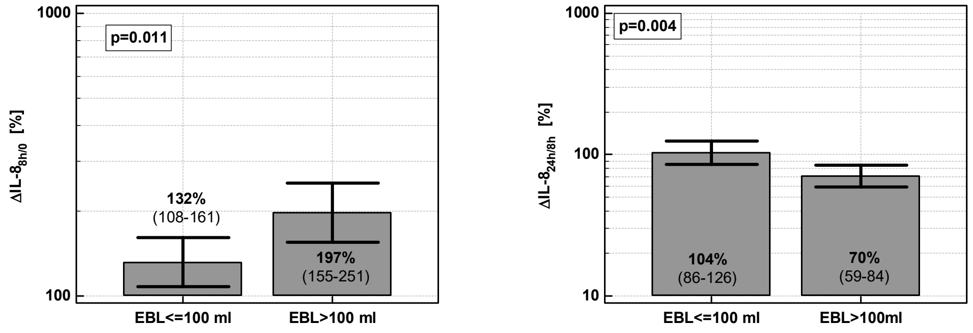

(**a**) (**b**)

Supplementary Figure S2. Effect of estimated blood loss (EBL) on percentage change in IL-8: (**a**) percentage change between 8h post incision and preoperative chemokine level ($\Delta IL\text{-}8_{8h/0}$); (**b**) percentage change between 24h and 8h post incision ($\Delta IL\text{-}8_{24h/8h}$). Data presented as geometric means with 95%*CI* and analyzed using t-test for independent samples.
