# Supplementary material for: Distinct Chemokine Dynamics in Early Postoperative Period after Open and Robotic Colorectal Surgery
Source: J Clin Med. 2019 Jun 19;8(6):879. doi: 10.3390/jcm8060879 (PMC6616914; doi:10.3390/jcm8060879)
Supplement: Supplementary file 1 [file jcm-08-00879-s001.zip › SupFig3.pdf]

Supplementary Figure S3

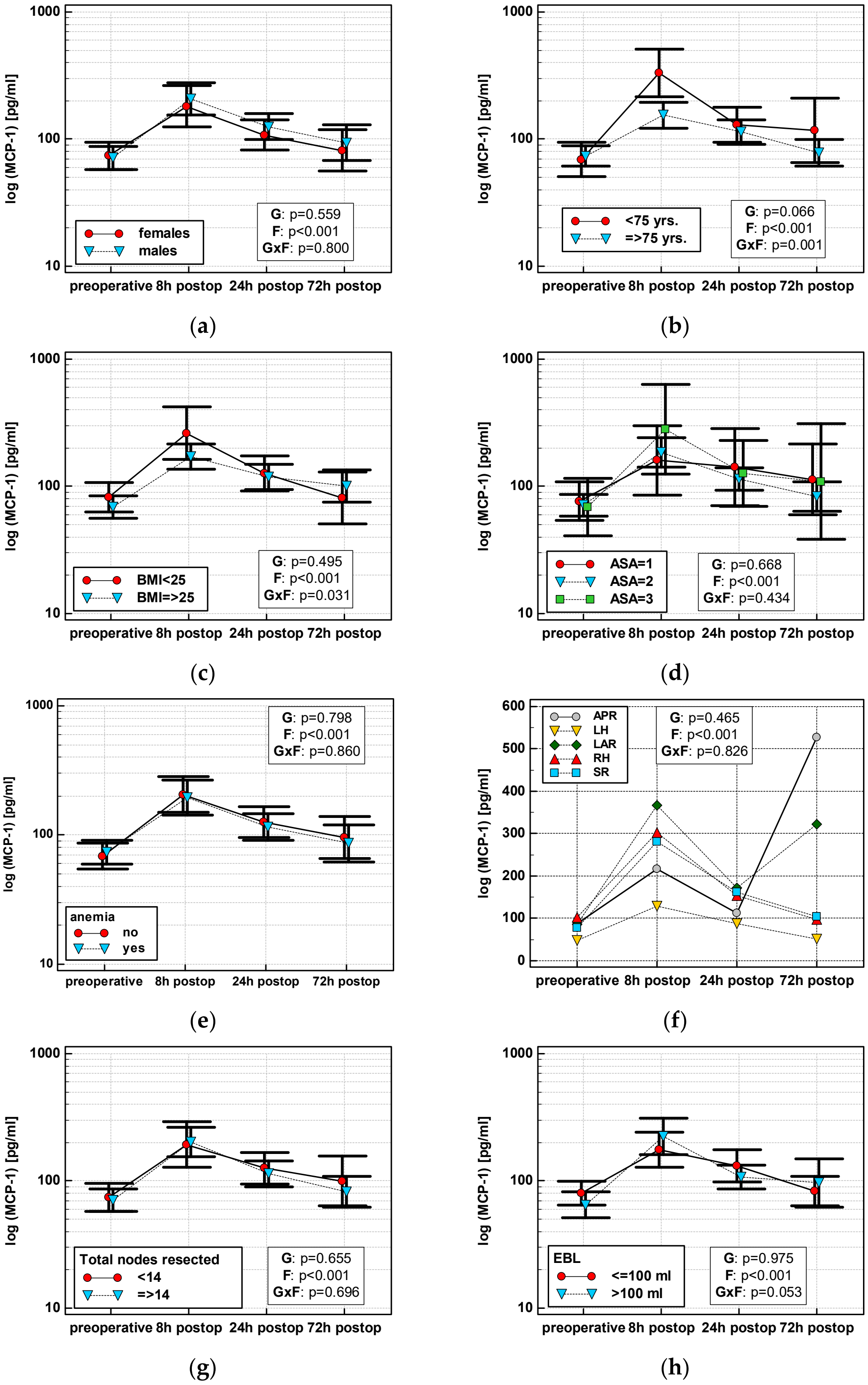

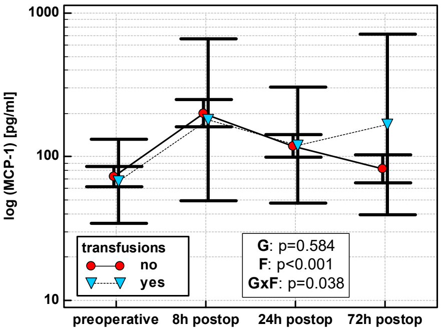

(**i**)

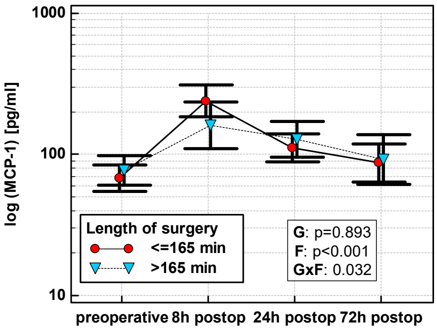

(**j**)

Supplementary Figure S3. Effect of various clinical parameters on perioperative MCP-1 dynamics: (**a**) sex; (**b**) age; (**c**) BMI; (**d**) physical status classification system (ASA); (**e**) anemia; (**f**) surgical procedure; (**g**) total number of resected lymph nodes; (**h**) estimated blood loss (EBL); (**i**) transfusions; (**j**) length of surgery. Data analyzed using two-way ANOVA with repeated measures on one factor. G, significance of group effect; F, significance of factor effect (time points); G×F, significance of interaction effect; APR, abdominoperineal resection; LAR, low anterior resection; RH, right hemicolectomy; LH; left hemicolectomy; SR, sigmoid resection.
