# Supplementary material for: Distinct Chemokine Dynamics in Early Postoperative Period after Open and Robotic Colorectal Surgery
Source: J Clin Med. 2019 Jun 19;8(6):879. doi: 10.3390/jcm8060879 (PMC6616914; doi:10.3390/jcm8060879)
Supplement: Supplementary file 1 [file jcm-08-00879-s001.zip › SupFig4.pdf]

Supplementary Figure S4

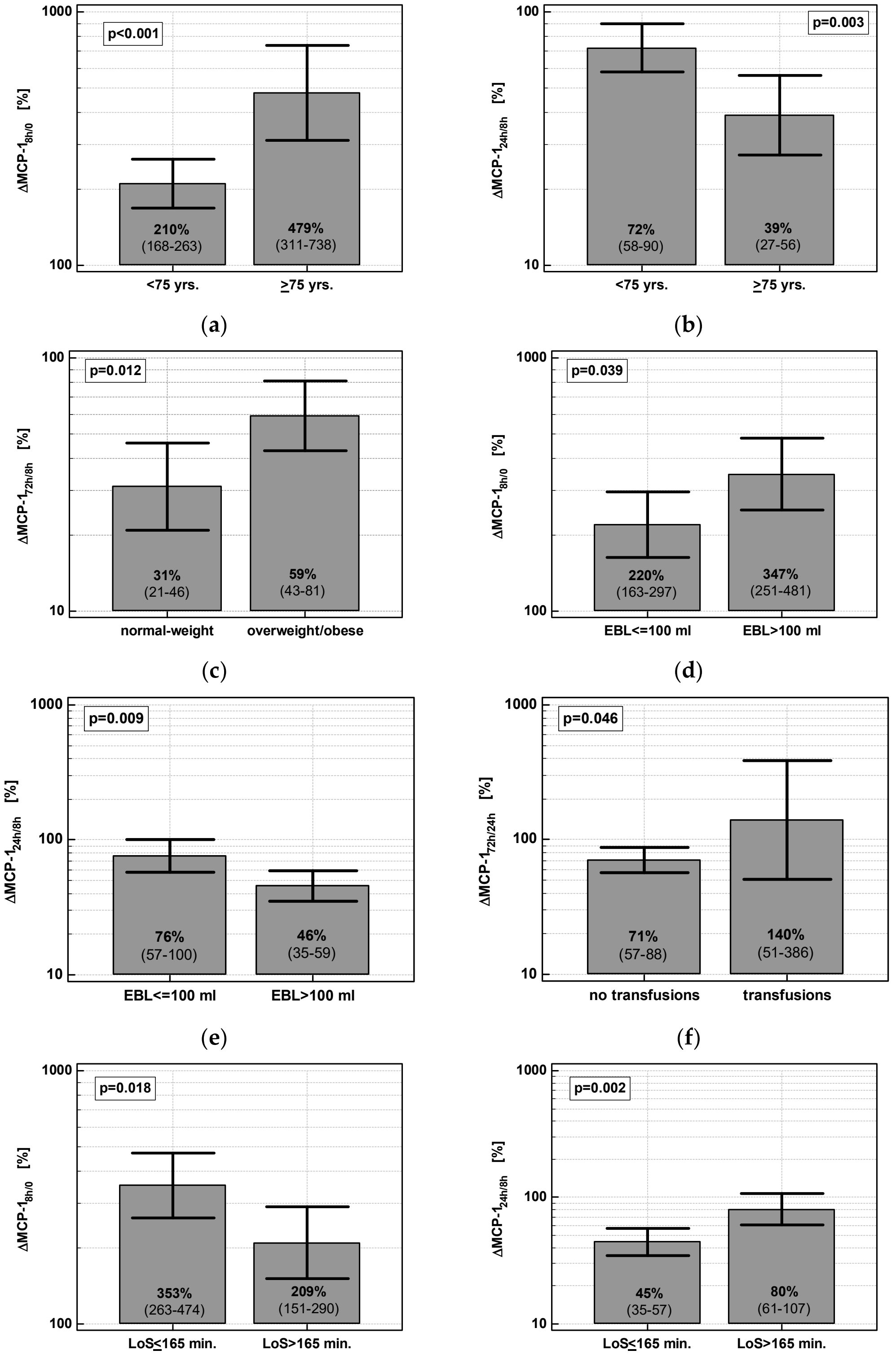

Supplementary Figure S4. Effect of various clinical parameters on percentage change in MCP-1: (**a**) patients′ age on ΔMCP-$1_{8h/0}$; (**b**) patients′ age on ΔMCP-$1_{24h/8h}$; (**c**) patient′s BMI on ΔMCP-$1_{72h/8h}$; (**d**) estimated blood loss (EBL) on ΔMCP-$1_{8h/0}$; (**e**) estimated blood loss (EBL) on ΔMCP-$1_{24h/8h}$; (**f**) transfusions on ΔMCP-$1_{72h/24h}$; (**g**) length of surgery (LoS) on ΔMCP-$1_{8h/0}$; (**h**) length of surgery (LoS) on ΔMCP-$1_{24h/8h}$. Data presented as geometric means with 95%*CI* and analyzed using t-test for independent samples.
