# Supplementary material for: Distinct Chemokine Dynamics in Early Postoperative Period after Open and Robotic Colorectal Surgery
Source: J Clin Med. 2019 Jun 19;8(6):879. doi: 10.3390/jcm8060879 (PMC6616914; doi:10.3390/jcm8060879)
Supplement: Supplementary file 1 [file jcm-08-00879-s001.zip › SupFig6.pdf]

Supplementary Figure S6

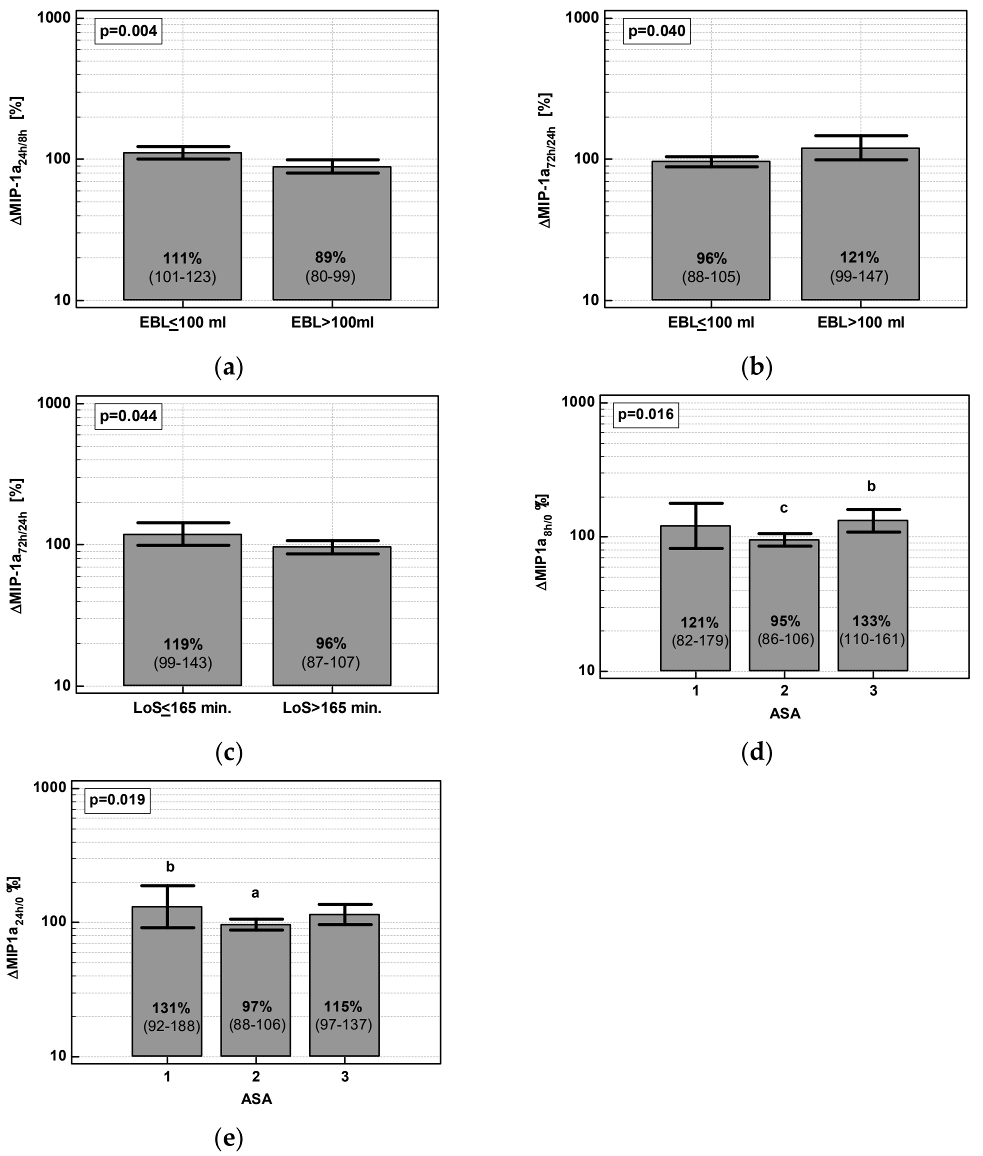

Supplementary Figure S6. Effect of various clinical parameters on percentage change in MIP-1α: (**a**) estimated blood loss (EBL) on ΔMIP-1$\alpha_{24h/8h}$; (**b**) estimated blood loss (EBL) on ΔMIP-1$\alpha_{72h/24h}$; (**c**) length of surgery (LoS) on ΔMIP-1$\alpha_{72h/24h}$; (**d**) physical status classification system (ASA) score on ΔMIP-1$\alpha_{8h/0}$; (**e**) physical status classification system (ASA) score on ΔMIP-1$\alpha_{24h/0}$;. Data presented as geometric means with 95%*CI* and analyzed using t-test for independent samples (panels a-c) or one-way ANOVA (panels d-e). a, significantly different from ASA=1; b, significantly different from ASA=2; c, significantly different from ASA=3.
